# Supplementary material for: In mouse chronic pancreatitis CD25+FOXP3+ regulatory T cells control pancreatic fibrosis by suppression of the type 2 immune response
Source: Nat Commun. 2022 Aug 3;13:4502. doi: 10.1038/s41467-022-32195-2 (PMC9349313; doi:10.1038/s41467-022-32195-2)
Supplement: Supplementary file 1 — Supplementary Information [file 41467_2022_32195_MOESM1_ESM.pdf]

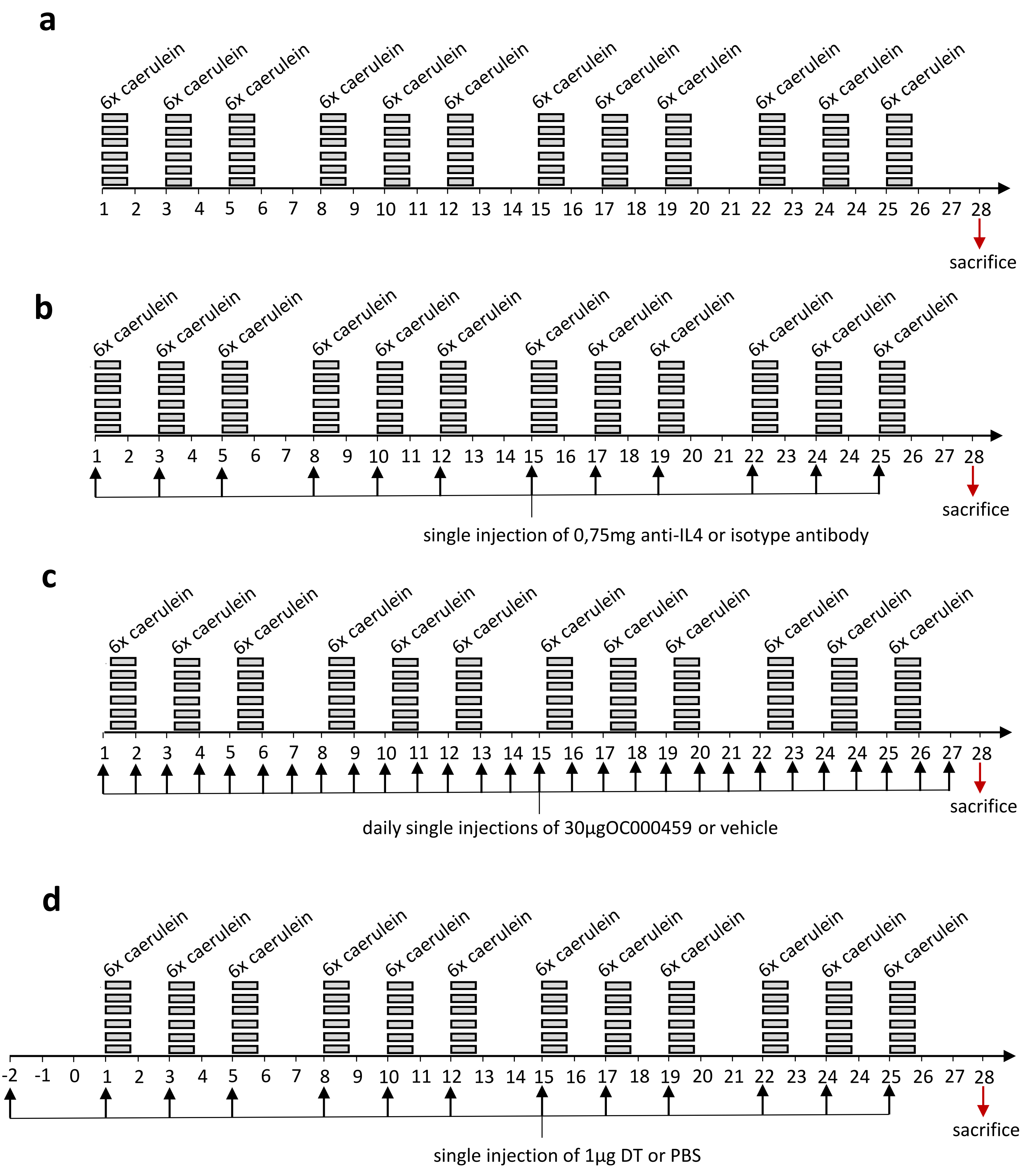

Supplementary Figure 1: Injection and treatment scheme of mouse chronic pancreatitis models. a The scheme illustrates the design of the experimental animal studies, the induction of chronic pancreatitis, **b** the treatment with neutralizing IL-4 antibody, **c** the treatment with the CRTH2 antagonist OC000459 and **d** the depletion of regulatory T cells over the course of CP.

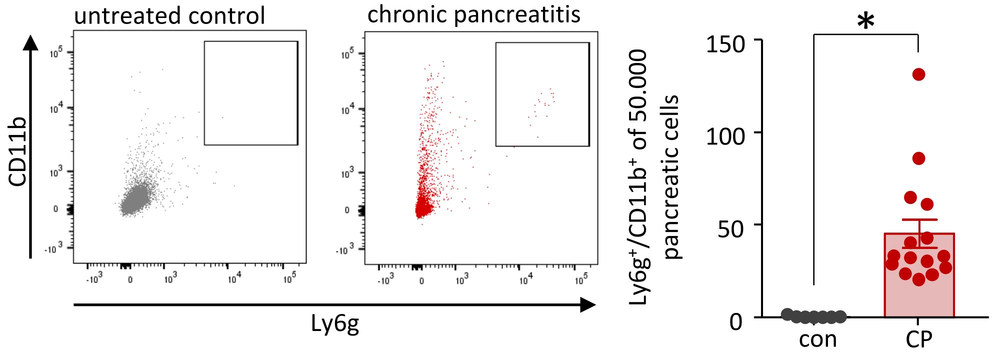

Supplementary Figure 2: Neutrophil infiltration during chronic pancreatitis. CD11b$^+$/Ly6g$^+$ neutrophil granulocytes showed a significant increase during CP, but the total amount of infiltrating neutrophils is negligible (*p*=0.0009, con n=7/CP n=15). All data were presented as means ± SEM, statistically significant differences were tested by unpaired two tailed students t-test for independent samples and significance levels of p<0.05 are marked by an asterisk.

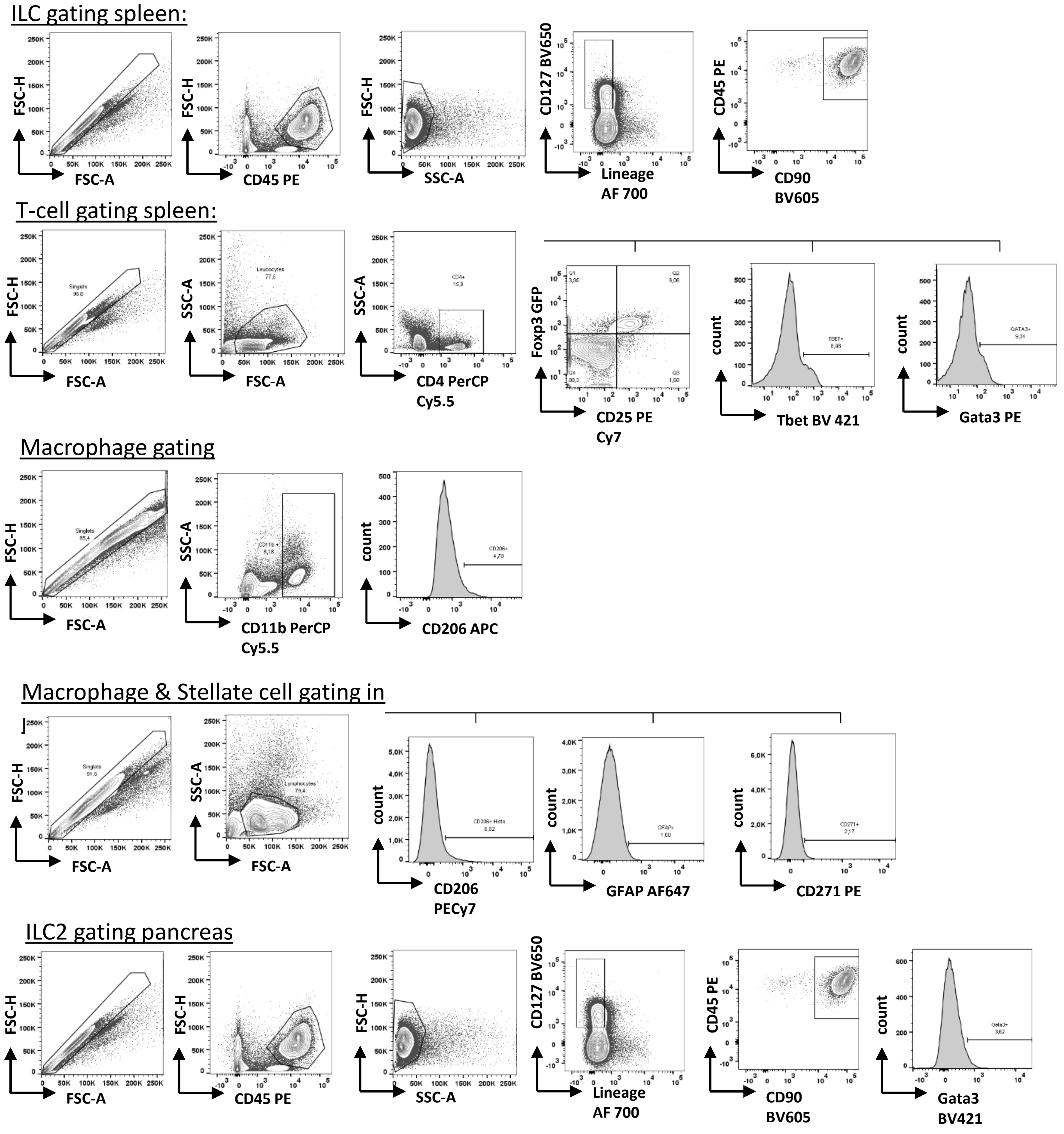

Supplementary Figure 3: Gating strategy of flow cytometry analysis from spleen and pancreas tissue. Gating strategy for innate lymphoid cells, T-cells, macrophages and stellate cells in spleen and pancreas.

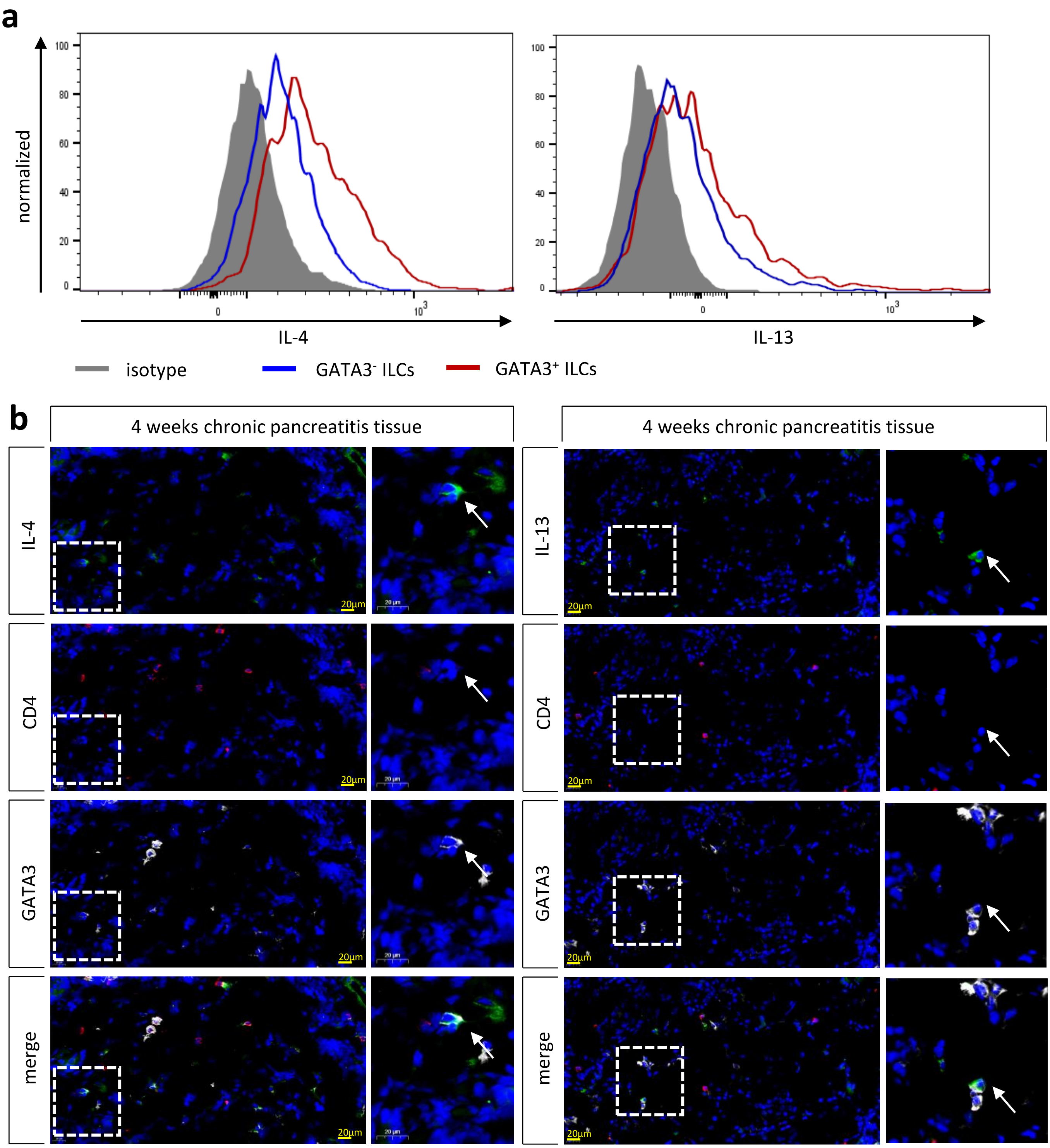

Supplementary Figure 4: ILC2s are source of IL-4/IL-13. Splenocytes of mice were isolated and analyzed by flow cytometry. The intracellular cytokine production of ILCs was detected by labeling for IL-4 and IL-13 after brefeldin a/monensin treatment. ILC2s were identified by the expression of GATA3. **a** The mean fluorescent intensity of IL-4 and IL-13 staining is elevated in GATA3^{+} ILCs (ILC2s) compared to GATA3^{-} ILCs or isotype controls. **b** Immunofluorescent labelling of GATA3^{+}/CD4^{-} cells in CP tissue of mice detected co-localization with the type 2 cytokines IL-4 and IL-13, scale bars represent 20µm. The results shown were repeated in 4 independent mouse sample sets with the same result (**a**).

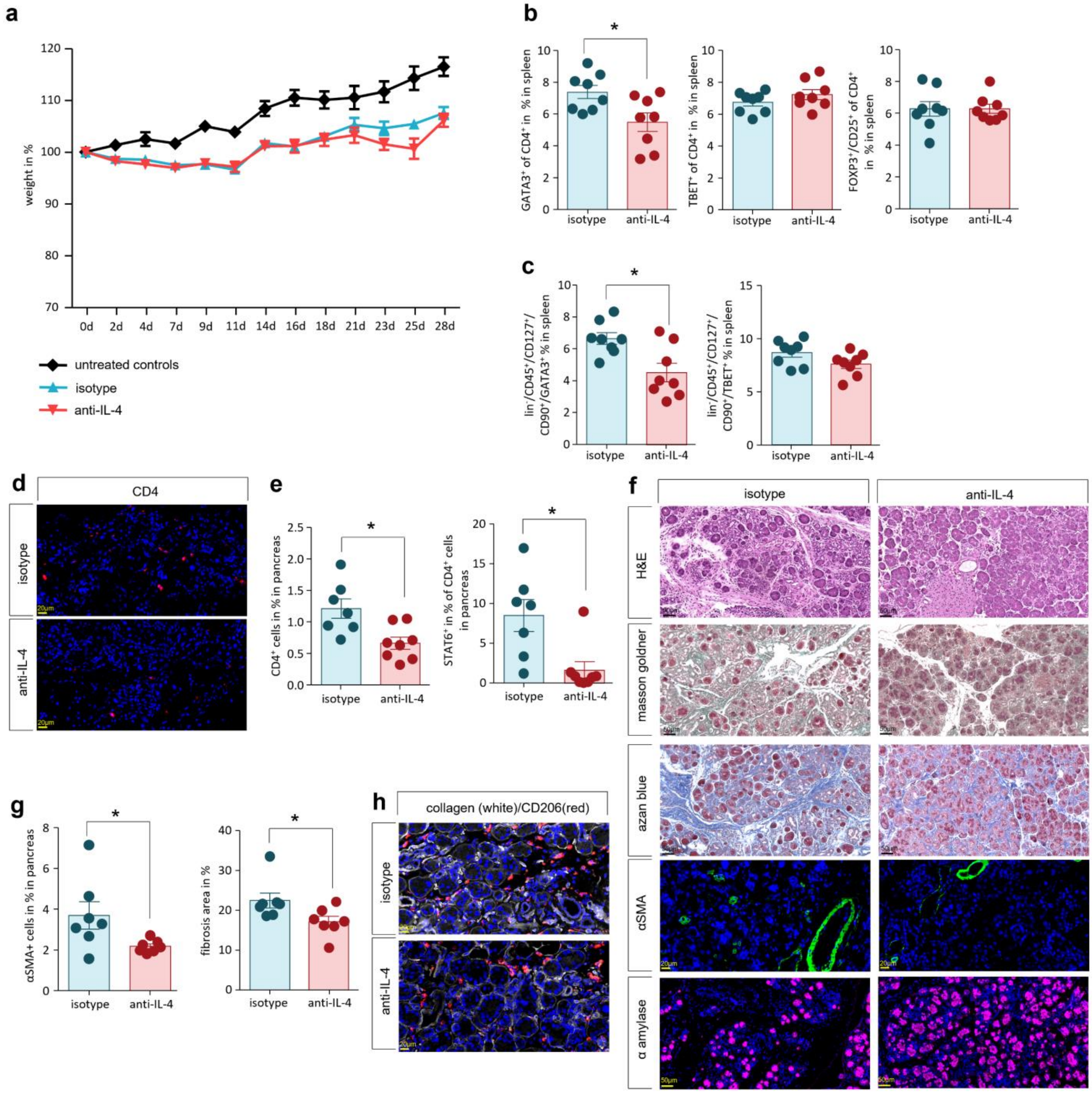

Supplementary Figure 5: The effect of an anti-IL-4 treatment in a mouse model of chronic pancreatitis. CP was induced in C57Bl/6 mice by repetitive caerulein injections over 4 weeks. In addition, animals received 0,75mg of anti-IL-4 antibody or isotype control 3 times a week. **a** Body weight changes over time were not significant between all groups (untreated controls n=4, isotype n=8, anti-IL-4 n=8). **b** T cell ($CD4^+$) differentiation in spleen is shown by intracellular transcription factor staining of GATA3 for Th2 cells (*p*=0.0192, isotype n=8, anti-IL-4 n=8), of TBET for Th1 cells (isotype n=8, anti-IL-4 n=8) and of FOXP3 in combination with CD25 to discriminate Treg cells (isotype n=8, anti-IL-4 n=8). **c** ILCs ($lin^-/CD45^+/CD127^+/CD90^+$) were discriminated by the expression of TBET (ILC1s) (isotype n=8, anti-IL-4 n=8) and GATA3 (ILC2s) (*p*=0.0083, isotype n=8, anti-IL-4 n=8) in spleen. **d, e** Anti-IL-4 antibody treatment significantly reduced the $CD4^+$ T cell infiltration of the pancreas in CP mice (*p*=0.0080, isotype n=7, anti-IL-4 n=8), scale bars represent 20µm. **e** $CD4^+/STAT6^+$ cells were nearly absent in CP tissue of anti-IL-4 treated mice (*p*=0.0080, isotype n=7, anti-IL-4 n=8). **f** Reduced tissue fibrosis was observed in anti-IL-4 treated animals by H&E, Masson Goldner trichrome and azan blue staining, scale bars represent 50µm. Decreased number of αSMA^+ cells and increased number of acinar cells underline the diminished tissue destruction in anti-IL-4 treated mice, scale bars represent 20 µm and 50µm. **g** Quantification by pattern quant software revealed significantly less fibrotic tissue (*p*=0.0433, isotype n=7, anti-IL-4 n=7) and less αSMA^+ PSCs (*p*=0.0448, isotype n=7, anti-IL-4 n=7) in anti-IL-4 treated mice. **h** Immunofluorescence labelling of CD206 and collagen 1 showed less collagen in the pancreas, whereas the number of $CD206^+$ cells was not significantly affected. All data were presented as means ± SEM, statistically significant differences were tested by unpaired two tailed students t-test for independent samples and significance levels of p<0.05 are marked by an asterisk (**b, c, e, g**).

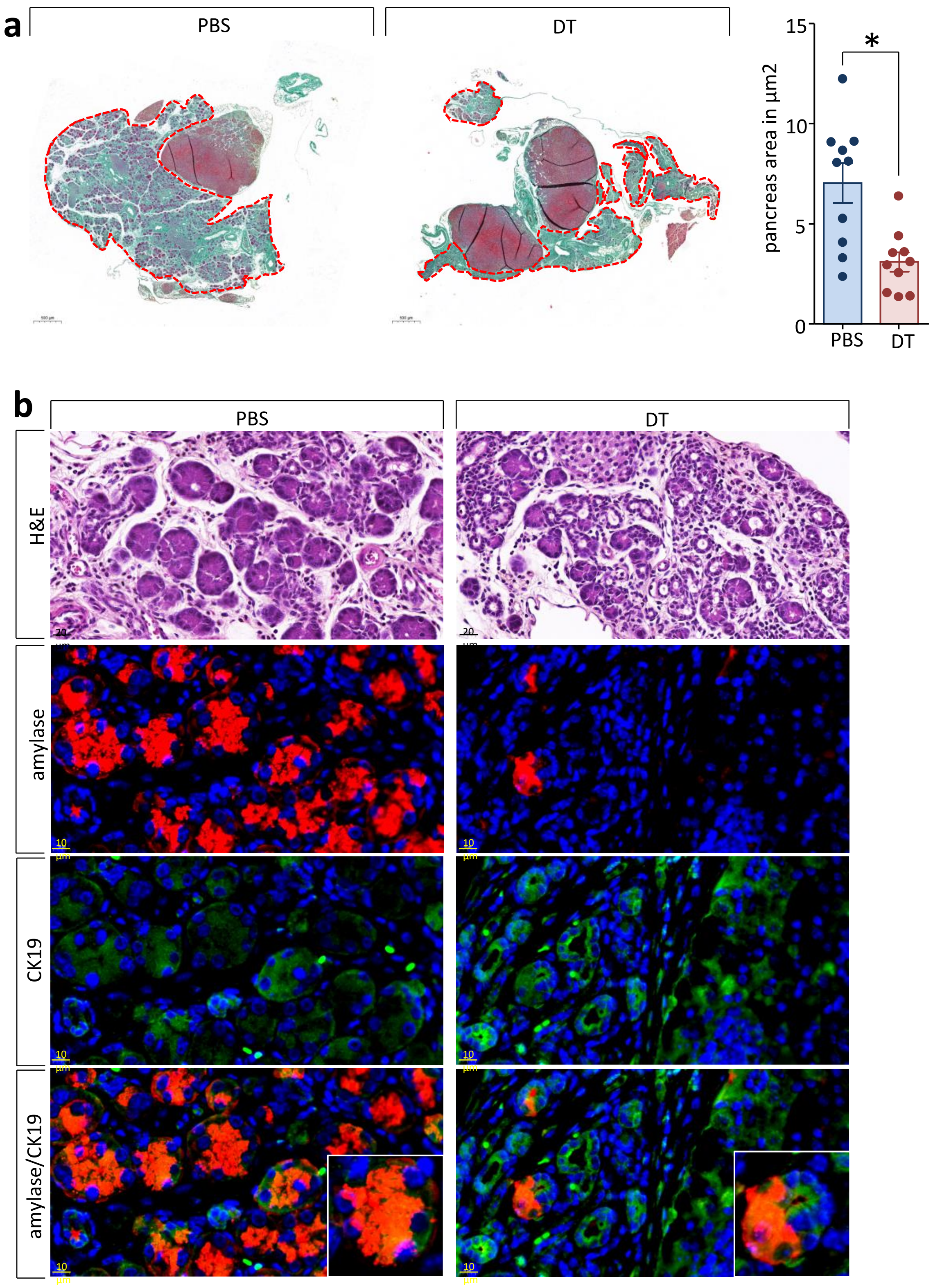

Supplementary Figure 6: Pancreas exocrine and endocrine tissue loss in DT treated DEREG mice. Treg depletion results in a decrease of exocrine pancreatic tissue shown by Masson Goldner staining. **a** The area containing exocrine tissue is significantly reduced in the DT-treated group (*p*=0.0022, PBS n=10/DT n=10), scale bars represent 500µm. **b** H&E staining of CP tissue showed the remarkable occurrence of acinar to ductal metaplasia in DT-treated mice. Labeling of α-amylase and cytokeratin 19 (CK19) underlines this observation and showed a reduction of exocrine tissue, whereas ductal structures without secretory compartment dominate, scale bars represent 20µm or 10µm. All data were presented as means ± SEM, statistically significant differences were tested by unpaired two tailed students t-test for independent samples and significance levels of p<0.05 are marked by an asterisk (**a**).

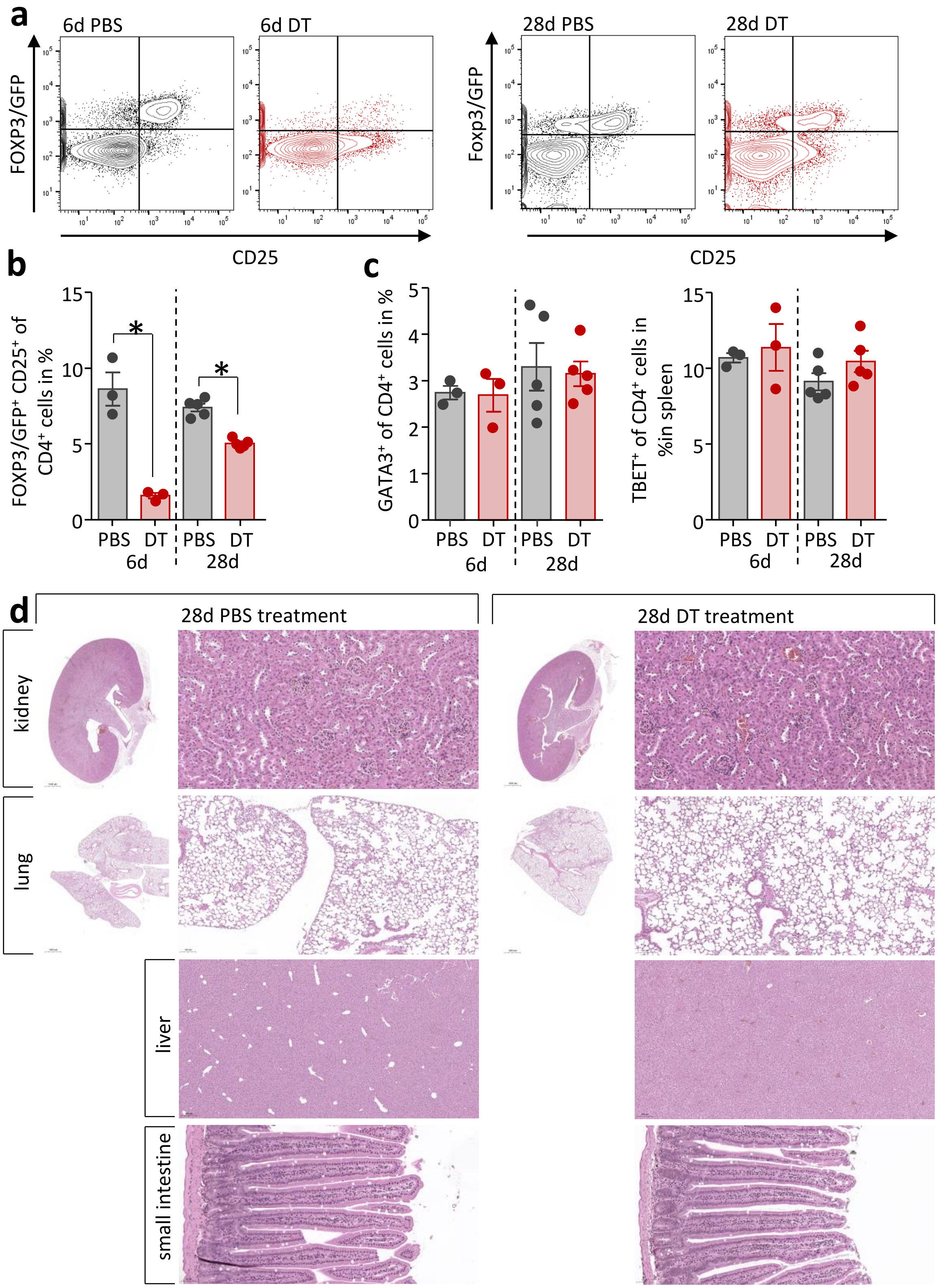

Supplementary Figure 7: The systemic effect of Treg depletion over time in mice. Long-term depletion of Treg cells in DEREG mice was performed over 28d without the induction of CP by caerulein. **a, b** The splenic T cell response was evaluated by flow cytometry at day 6 and 28 after starting the continuous depletion. $GFP^+/CD25^+$ Treg cells were completely absent after 6d (p=0.0032, PBS n=3/DT n=3), but a small resistant population of $GFP^+/CD25^+$ Treg cells has re-appeared at day 28 (p<0.0001, PBS n=5/DT n=5). **c** The percentage of Th1/Th2 cells did not change 6d (PBS n=3/DT n=3), or 28d (PBS n=5/DT n=5) in the absence of Treg cells. **d** Kidney, lung, liver and small intestine were examined 28d after continuous Treg depletion but did not show any morphological changes or alterations compared to PBS treated controls, scale bars represent 1000-50µm. All data were presented as means ± SEM, statistically significant differences were tested by unpaired two tailed students t-test for independent samples and significance levels of p<0.05 are marked by an asterisk (**b**).

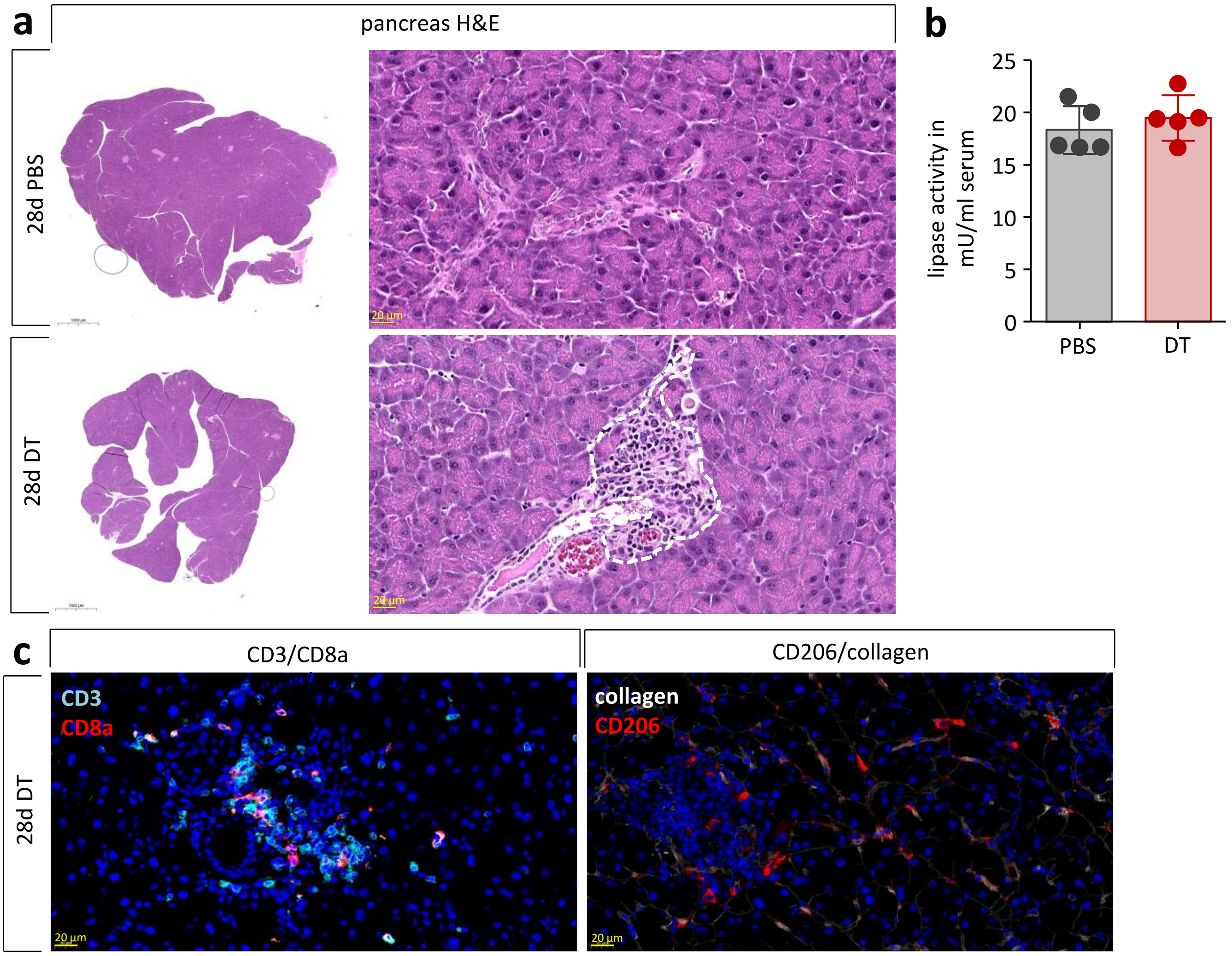

Supplementary Figure 8: The effect of Treg depletion on the pancreas over time in mice. Pancreatic tissue was analyzed after long-term depletion of Treg cells in DEREG mice without the induction of CP with caerulein. **a** H&E staining of the pancreas showed isolated areas of infiltrate in 2 of 5 animals, scale bars represent 1000-50µm. However, the exocrine and endocrine parts of the organ were completely intact and showed no changes or signs of an acute inflammatory reaction. **b** This was confirmed by analysis of the serum lipase activity, which showed no difference to the PBS treated group (PBS n=5/DT n=5). **c** Further examination of the infiltrates gave evidence that they were mainly containing $CD3^{+}$ and $CD8\alpha^{+}$ T cells. No increase of CD206 alternatively activated macrophages and no increased deposition of collagen 1 could be observed in these areas, scale bars represent 20µm. All data were presented as means ± SEM.
