# Supplementary material for: In mouse chronic pancreatitis CD25+FOXP3+ regulatory T cells control pancreatic fibrosis by suppression of the type 2 immune response
Source: Nat Commun. 2022 Aug 3;13:4502. doi: 10.1038/s41467-022-32195-2 (PMC9349313; doi:10.1038/s41467-022-32195-2)
Supplement: Supplementary file 3 — Reporting Summary [file 41467_2022_32195_MOESM3_ESM.pdf]

nature research

Corresponding author(s): Matthias Sendler

Last updated by author(s): May 24, 2022

Reporting Summary

Nature Research wishes to improve the reproducibility of the work that we publish. This form provides structure for consistency and transparency in reporting. For further information on Nature Research policies, see our Editorial Policies and the Editorial Policy Checklist.

Statistics

For all statistical analyses, confirm that the following items are present in the figure legend, table legend, main text, or Methods section.

n/a	Confirmed	
☐	☒	The exact sample size (*n*) for each experimental group/condition, given as a discrete number and unit of measurement
☐	☒	A statement on whether measurements were taken from distinct samples or whether the same sample was measured repeatedly
☐	☒	The statistical test(s) used AND whether they are one- or two-sided<br>*Only common tests should be described solely by name; describe more complex techniques in the Methods section.*
☐	☒	A description of all covariates tested
☐	☒	A description of any assumptions or corrections, such as tests of normality and adjustment for multiple comparisons
☐	☒	A full description of the statistical parameters including central tendency (e.g. means) or other basic estimates (e.g. regression coefficient) AND variation (e.g. standard deviation) or associated estimates of uncertainty (e.g. confidence intervals)
☐	☒	For null hypothesis testing, the test statistic (e.g. *F*, *t*, *r*) with confidence intervals, effect sizes, degrees of freedom and *P* value noted<br>*Give P values as exact values whenever suitable.*
☒	☐	For Bayesian analysis, information on the choice of priors and Markov chain Monte Carlo settings
☒	☐	For hierarchical and complex designs, identification of the appropriate level for tests and full reporting of outcomes
☒	☐	Estimates of effect sizes (e.g. Cohen's *d*, Pearson's *r*), indicating how they were calculated

Our web collection on statistics for biologists contains articles on many of the points above.

Software and code

Policy information about availability of computer code

Data collection	The data were not collected by using a specifc software.
Data analysis	The statistical evaluation of the experiments were carried out in GraphPad Prism (V5.04) and SigmaPlot (11.0). Flow cytometry date were analysed with BD FACS DIVA (v9.0) Software and FlowJo (V10). The quantification of histological stainings were performed by using Sysmex Quant Center software (2.2.1.88915).<br>Microarray data analysis was performed using the Rosetta Resolver software system including Ratio Builder software (Version 7.2).<br>Heatmap was created by using RStudio (V3.6.1, library: gplost and RColorBrewer).

For manuscripts utilizing custom algorithms or software that are central to the research but not yet described in published literature, software must be made available to editors and reviewers. We strongly encourage code deposition in a community repository (e.g. GitHub). See the Nature Research guidelines for submitting code & software for further information.

Data

Policy information about availability of data

All manuscripts must include a data availability statement. This statement should provide the following information, where applicable:

- Accession codes, unique identifiers, or web links for publicly available datasets
- A list of figures that have associated raw data
- A description of any restrictions on data availability

Microarray data have been deposited in the National Center for Biotechnology Information (NCBI) Gene Expression Omnibus (GEO) database and are accessible through the following GEO accession number: GSE192517 [https://www.ncbi.nlm.nih.gov/geo/query/acc.cgi?acc=GSE192517]. The authors declare that all data supporting the findings of this study are available within the paper and its Supplementary Information files. Source data are provided with this paper.

Field-specific reporting

Please select the one below that is the best fit for your research. If you are not sure, read the appropriate sections before making your selection.

- [x] Life sciences
- [ ] Behavioural & social sciences
- [ ] Ecological, evolutionary & environmental sciences

For a reference copy of the document with all sections, see nature.com/documents/nr-reporting-summary-flat.pdf

Life sciences study design

All studies must disclose on these points even when the disclosure is negative.

Sample size	The sample size for all animal experiments were determined by usage of power estimation using GPower (V 3.1). All experiments using cells were performed without sample size calculation, based on the experience and previous works (Gastroenterology. 2018 Feb;154 (3):704-718.e10., Gastroenterology. 2015 Sep;149(3):765-76.e10., Gastroenterology. 2020 Jan;158(1):253-269.e14.). At least a minimum of 3 independent samples each group were analysed.
Data exclusions	No sample was excluded from animal experiments.
Replication	The chronic pancreatitis model (4 weeks Caerulein treatment, PBS vs Diphteria toxin application) was repeated three times, on the one hand to check the results, on the other hand to obtain enough material for the different analyses. All replications show the same result.<br>The treatment of mice with the CRTH2 antagonist OC000459 and neutralizing IL-4 antibody were performed in 8 animals/group.<br>All experiments involvings cells were repeated at least minimum 3 times as indicated in the figure legens. All replicate experiments produce consistent data.
Randomization	No randomization was used for the experiments. Animal studies based on repeated applications of different treatments (Diphteria Toxin, Anti-IL-4 or OC000459) could not be randomized. However, before the start of the experiment, the animals were randomly assigned to the respective treatment groups. Further flow cytometry analysis or histological analysis were performed in a blinded manner.
Blinding	The evaluations of the flow cytometry analyses as well as the histological quantifications were performed in a blinded manner. The groups were blinded for the investigators during analysis.

Reporting for specific materials, systems and methods

We require information from authors about some types of materials, experimental systems and methods used in many studies. Here, indicate whether each material, system or method listed is relevant to your study. If you are not sure if a list item applies to your research, read the appropriate section before selecting a response.

Materials & experimental systems

n/a	Involved in the study	
[ ]	[x]	Antibodies
[x]	[ ]	Eukaryotic cell lines
[x]	[ ]	Palaeontology and archaeology
[ ]	[x]	Animals and other organisms
[ ]	[x]	Human research participants
[x]	[ ]	Clinical data
[x]	[ ]	Dual use research of concern

Methods

n/a	Involved in the study	
[ ]	[x]	ChIP-seq
[ ]	[x]	Flow cytometry
[x]	[ ]	MRI-based neuroimaging

Antibodies

Antibodies used

The following antibodies were used for immunofluorescence:
anti-GFP (ab6673, abcam)
anti-CD3 (100202, BioLegend)
anti-Mrc1/CD206 (OASA05048, aviva-sysbio)
anti-αSMA (M0851, Dako)
anti-CD90 (14-0900-85, Invitrogen)
anti-Amphiregulin (sc-5796, SanatCruz)
anti-Amphiregulin (PA5-27298, Invitrogen)
anti-collagen I (ab34710, abcam)
anti-amylase (sc-46657, Santa Cruz)
anti-Ki-67 (IHC-00375, Bethyl)
anti-FGF Receptor (9740S, cell signaling)
anti-InhibinβA (C9B1223, BioGenesis)
anti-Cytokeratin 19 (ab15463, abcam)

anti-CD4 (UM800010, origene)
anti-IL13 (bs-0560R, Bioss Antibodies)
anti-IL33 (AF3626, R&D systems)
anti-STAT6 (ab32520, abcam)
anti-Gata3 (14-9966-80, eBioscience)

secondary antibodys:
anti-goat-Cy3 (705-165-147, Jackson ImmunoResearch)
anti-mouse-FITC (115-095-146, Jackson ImmunoResearch)
anti-mouse-Cy3 (115-165-166, Jackson ImmunoResearch)
anti-mouse-Cy5 (115-175-146, Jackson ImmunoResearch)
anti-rabbit-FITC (711-095-152, Jackson ImmunoResearch)
anti-rabbit-Cy3 (111-165-144, Jackson ImmunoResearch)
anti-rat-Cy3 (112-165-062, Jackson ImmunoResearch)
AlexaFluor647 donkey anti-rabbit (A31573, Invitrogen)

The following antibodies were used for Flow-cytometry analysis:
anti-CD25-PE/Cy7 (102016, BioLegend)
anti-CD25-PE/Cy7 (302612, BioLegend)
anti-CD4-PerCP/Cy5.5 (100433, BioLegend)
anti-CD4-PE (100408, BioLegend)
anti-CD4-PerCP/Cy5.5 (100434, BioLegend)
anti-CD4-Brilliant-Violet510™ (116026, BioLegend)
anti-CD4-AlexaFluor488 (317420, BioLegend)
anti-CD8α-PE/Cy5 (100710, BioLegend)
anti-CD8α-PE (100708, BioLegend)
anti-Tbet-Brilliant Violet 421 (644815, BioLegend)
anti-Tbet-PerCP/Cy5.5 (644806, BioLegend)
anti-Tbet-Brilliant Violet605™ (644817, BioLegend)
anti-GATA3-PE (653803, BioLegend)
anti-GATA3-PE (130-100-652, MiltenyiBiotec)
anti-Gata3-Brilliant Violet421 (653814, BioLegend)
anti-Rorγt-APC (130-123-840, MiltenyiBiotec)
anti-FoxP3-AlexaFluor488 (53-4774-42, Invitrogen)
anti-FoxP3-AlexaFluor647 (320114, BioLegend)
anti-PTGDR2/CD294-AlexaFlour594 (C47774-af594, SAB Signalway Antibody)
anti CD69-Brilliant Violet510 (104532, BioLegend)
anti-CD69-Brilliant Violet510™ (310936, BioLegend)
anti-CD11b-PerCP/Cy5.5 (101228, BioLegend)
anti-CD206-APC (141708, BioLegend)
anti-CD206-PE/Cy7 (141719, BioLegend)
anti-CD163-PE (12-1631-82, Invitrogen)
anti-CD163-PE/Dazzle (155315, BioLegend)
anti-Ly6g-BV421 (127628, BioLegend)
anti-lin-AlexaFlour700 (77923, BioLegend)
anti-CD45-PerCP (103130, BioLegend)
anti-CD45-PE (103106, BioLegend)
anti-CD45-PE/Cy5 (103109, BioLegend)
anti-CD90-Brilliant Violet605 (105343, BioLegend)
anti-CD127-Brilliant Violet650 (135043, BioLegend)
anti-GFAP-Alexa Fluor647 (51-9792-82, Invitrogen)
anti-CD271-PE (12-9400-42, Invitrogen)
anti-CCR2-FITC (150608, BioLegend)
anti-IL4-AlexaFluor488 (504109, BioLegend)
anti-IL13-PE (159403, BioLegend)
anti-IFNγ-Brilliant Violet650™(505831, BioLegend)
anti-TNFα-PE/Cy7 (506324, BioLegend)
anti-IL10-APC (505010, BioLegend) PE/Cyanine7

Isotype antibodies:
Rat IgG2b, Isotype Ctrl Antibody (400617, BioLegend)
Brilliant Violet 510™ Armenian Hamster IgG Isotype Ctrl Antibody (400941, BioLegend)
Brilliant Violet 421™ Mouse IgG2b, Isotype Ctrl Antibody (400341, BioLegend)
Brilliant Violet 421™ Mouse IgG1, Isotype Ctrl Antibody (400157, BioLegend)
Brilliant Violet 650™ Rat IgG1, Isotype Ctrl Antibody (400437, BioLegend)
PerCP/Cy5.5 mouse IgG1, Isotype Ctrl Antibody (400150, BioLegend)
IgG1 Antibody, anti-mouse APC (130-117-099, MiltenyiBiotec)
AlexaFluor488 Rat IgG1, Isotype Ctrl (400417, BioLegend)

PE Mouse IgG2b, Isotype Ctrl (400313, BioLegend)
PE Mouse IgG1, Isotype Ctrl (551436, BD)
APC Rat IgG2b, Isotype Ctrl (400612, BioLegend)
PE/Cyanine7 Rat IgG2b, Isotype Ctrl Antibody (400617, BioLegend)

neutralizing anti-IL-4 antibody treatment:
Ultra-LEAF™ Purified anti-mouse IL-4 antibody (504138, BioLegend)
Ultra-LEAF™ Purified Rat IgG1 isotype (400462, BioLegend)

Validation

All antibodies were comercial available and characterized by manufacturers. All antibodies which were used for flow cytometry analysis were characterized and evaluated in previouse projekts (Sendler, M. et al. NLRP3 Inflammasome Regulates Development of Systemic Inflammatory Response and Compensatory Anti-Inflammatory Response Syndromes in Mice With Acute Pancreatitis. Gastroenterology 158, 253-269.e14 (2020). or Glaubitz, J. et al. Experimental pancreatitis is characterized by rapid T cell activation, Th2 differentiation that parallels disease severity, and improvement after CD4+ T cell depletion. Pancreatology (2020)).

All primary antibodies which were used for histology were validated in positive controls or in previous projects (Sendler, M. et al. Cathepsin B-Mediated Activation of Trypsinogen in Endocytosing Macrophages Increases Severity of Pancreatitis in Mice Gastroenterology. 2018 Feb;154(3):704-718.e10. or Sendler, M. et al. Complement Component 5 Mediates Development of Fibrosis, via Activation of Stellate Cells, in 2 Mouse Models of Chronic Pancreatitis Gastroenterology. 2015 Sep;149(3):765-76.e10.)

Animals and other organisms

Policy information about studies involving animals; ARRIVE guidelines recommended for reporting animal research

Laboratory animals

Male C57Bl/6J mice were purchased from Charles River Laboratories (Sulzfeld, Germany). The DEREG mice (male and female) were breed and maintained in the central animal facility of the university medicine Greifswald.
All animals were used at an age of 8-12 weeks. Animals were kept under controlled housing conditions; 21–24°C (humidity 50-70%) and 12-h light/12-h dark cycle. All animals were inspected daily for their physical conditions. Animals which showing signs moderate pain or suffering were euthanized based on pre-determined human endpoints.

Wild animals

No wild animals were used

Field-collected samples

No field collected samples were used

Ethics oversight

All animal experiments were carried out after prior review and approval by the local animal welfare commission (Landesamt für Landwirtschaft, Lebensmittelsicherheit und Fischerei Mecklenburg-Vorpommern) (Lallf 7221.3-1-011/17 and Lallf 7221.3-1-048/14). All animal experiments were carried out in accordance with the Arrive guidelines and the 3R rules.

Note that full information on the approval of the study protocol must also be provided in the manuscript.

Human research participants

Policy information about studies involving human research participants

Population characteristics

Paraffin-embedded pancreatic tissue from patients with chronic pancreatitis was used. All patients underwent pancreatic resection due to complications of chronic pancreatitis, and pancreatic cancer was excluded by the pathologist.
Chronic pancreatitis patients (n=7) had a mean age of 55years (range from 39 to 85), male and female patients were included in the study. Healthy blood donors (n=10) were used as controls with an age >40 years, male as well as female were included.

Recruitment

Patients that were admitted to our clinic with a diagnosis of chronic pancreatitis were recruited into the study without any other bias.

Ethics oversight

Chronic pancreatitis EDTA blood samples were collected at the university medicine Greifswald after approval by the Ethical committee of the university medicine Greifswald (III UV 91/03). All patients gave written and informed consent. Control blood was drawn in EDTA from healthy blood donors who gave written and informed consent. This procedure was approved by the Ethical committee of the university medicine Greifswald (BB 014/14). Human chronic pancreatitis tissue samples was collected in the context of the ChroPac trial (ISRCTN38973832).

Note that full information on the approval of the study protocol must also be provided in the manuscript.

ChIP-seq

Data deposition

- [x] Confirm that both raw and final processed data have been deposited in a public database such as GEO.
- [ ] Confirm that you have deposited or provided access to graph files (e.g. BED files) for the called peaks.

Data access links
May remain private before publication.

https://www.ncbi.nlm.nih.gov/geo/query/acc.cgi?acc=GSE192517

Files in database submission

BMDM_Co_1.chp

BMDM_Co_2.chp
BMDM_Co_3.chp
BMDM_LPS_1.chp
BMDM_LPS_2.chp
BMDM_LPS_3.chp
BMDM_Acini_1.chp
BMDM_Acini_2.chp
BMDM_Acini_3.chp
BMDM_Acini+Naf_1.chp
BMDM_Acini+Naf_2.chp
BMDM_Acini+Naf_3.chp

Genome browser session (e.g. UCSC)

Provide a link to an anonymized genome browser session for "Initial submission" and "Revised version" documents only, to enable peer review. Write "no longer applicable" for "Final submission" documents.

Methodology

Replicates

We used BMDMs isolated from C57Bl/6 mice which were maintained in the presence of 20ng MCSF over 7d until they were confluent. Co-incubation was performed with freshly prepared acinar cells from C57Bl/6 mice over 6h +/- Nafamostat. BMDMs were carefully washed before RNA Isolation. Transcriptome analyses were performed using 3 individual RNA samples.

Sequencing depth

Describe the sequencing depth for each experiment, providing the total number of reads, uniquely mapped reads, length of reads and whether they were paired- or single-end.

Antibodies

Describe the antibodies used for the ChIP-seq experiments; as applicable, provide supplier name, catalog number, clone name, and lot number.

Peak calling parameters

Specify the command line program and parameters used for read mapping and peak calling, including the ChIP, control and index files used.

Data quality

Describe the methods used to ensure data quality in full detail, including how many peaks are at FDR 5% and above 5-fold enrichment.

Software

Describe the software used to collect and analyze the ChIP-seq data. For custom code that has been deposited into a community repository, provide accession details.

Flow Cytometry

Plots

Confirm that:

- [x] The axis labels state the marker and fluorochrome used (e.g. CD4-FITC).
- [x] The axis scales are clearly visible. Include numbers along axes only for bottom left plot of group (a 'group' is an analysis of identical markers).
- [x] All plots are contour plots with outliers or pseudocolor plots.
- [x] A numerical value for number of cells or percentage (with statistics) is provided.

Methodology

Sample preparation

To analyse splenocytes, spleen was taken and mashed through a 70µm cell strainer. Cells were washed with PBS and centrifuged at 300g for 6 minutes. To lyse erythrocytes pellet was resuspended in 1 ml lysis buffer for 5 minutes. After washing with PBS and centrifugation at 300g for 6 minutes cells were stained with fluorescence antibodies.
To analyse leucocytes and cells from pancreatic tissue, Pancreatic tissue was removed and immediately dissociated with the Multi Tissue Dissociation Kit 1 (130-110-201, MiltenyiBiotec).

Instrument

For all Flow cytometry analysis we used BD™ LSR II Flow Cytometer System.

Software

Flow cytometry was analysed by BD FACS DIVA Software and FlowJo.

Cell population abundance

No sorting experiments were performed.

Gating strategy

Gating strategy for innate lymphoide cells including ILC2s, T-cells, macrophages and pancreatic stellate cells is provided in the supplementary figure 3.

- [x] Tick this box to confirm that a figure exemplifying the gating strategy is provided in the Supplementary Information.
